# (Ethanol-κ*O*)[2-(4-hydroxy­phen­yl)quinoline-4-carboxyl­ato-κ*O*]triphenyl­tin(IV)

**DOI:** 10.1107/S1600536809012239

**Published:** 2009-04-08

**Authors:** Kong Mun Lo, Seik Weng Ng

**Affiliations:** a Department of Chemistry, University of Malaya, 50603 Kuala Lumpur, Malaysia

## Abstract

The Sn atom in the title mol­ecule, [Sn(C_6_H_5_)_3_(C_16_H_10_NO_3_)(C_2_H_6_O)], shows a *trans*-C_3_SnO_2_ trigonal bipyramidal coord­in­ation. Adjacent mol­ecules are linked by O—H⋯O and O—H⋯N hydrogen bonds into a two-dimensional array parallel to (100). The ethanol ligand is disordered, with two sites of equal occupancy being resolved for the ethyl group.

## Related literature

Triphenyl­tin carboxyl­ates are coordinately saturated, and do not generally afford adducts; for some unusual examples of adducts with oxygen-donor ligands, see: Ng & Kumar Das (1997 ▶). For reviews of the structural chemistry of organotin carboxyl­ates, see: Tiekink (1991 ▶, 1994 ▶).
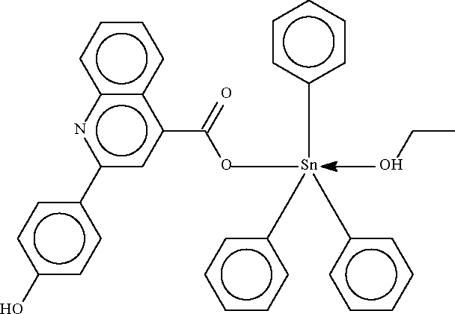


## Experimental

### 

#### Crystal data

[Sn(C_6_H_5_)_3_(C_16_H_10_NO_3_)(C_2_H_6_O)]
*M* *_r_* = 660.31
Monoclinic, 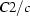

*a* = 38.9542 (5) Å
*b* = 9.7259 (2) Å
*c* = 17.8594 (3) Å
β = 116.632 (1)°
*V* = 6048.4 (2) Å^3^
*Z* = 8
Mo *K*α radiation
μ = 0.89 mm^−1^
*T* = 100 K
0.30 × 0.20 × 0.10 mm

#### Data collection

Bruker SMART APEX diffractometer
Absorption correction: multi-scan (*SADABS*; Sheldrick, 1996 ▶) *T*_min_ = 0.777, *T*_max_ = 0.917
23944 measured reflections
6937 independent reflections
5517 reflections with *I* > 2σ(*I*)
*R*_int_ = 0.038

#### Refinement

*R*[*F* ^2^ > 2σ(*F* ^2^)] = 0.036
*wR*(*F* ^2^) = 0.104
*S* = 1.26
6937 reflections
395 parameters
19 restraints
H atoms treated by a mixture of independent and constrained refinement
Δρ_max_ = 1.21 e Å^−3^
Δρ_min_ = −1.30 e Å^−3^

### 

Data collection: *APEX2* (Bruker, 2008 ▶); cell refinement: *SAINT* (Bruker, 2008 ▶); data reduction: *SAINT*; program(s) used to solve structure: *SHELXS97* (Sheldrick, 2008 ▶); program(s) used to refine structure: *SHELXL97* (Sheldrick, 2008 ▶); molecular graphics: *X-SEED* (Barbour, 2001 ▶); software used to prepare material for publication: *publlCIF* (Westrip, 2009 ▶).

## Supplementary Material

Crystal structure: contains datablocks global, I. DOI: 10.1107/S1600536809012239/tk2412sup1.cif

Structure factors: contains datablocks I. DOI: 10.1107/S1600536809012239/tk2412Isup2.hkl

Additional supplementary materials:  crystallographic information; 3D view; checkCIF report

## Figures and Tables

**Table 1:** Hydrogen-bond geometry (Å, °)

*D*—H⋯*A*	*D*—H	H⋯*A*	*D*⋯*A*	*D*—H⋯*A*
O3—H3⋯O2^i^	0.85 (1)	1.83 (2)	2.661 (3)	166 (6)
O4—H4⋯N1^ii^	0.84 (1)	1.95 (1)	2.789 (4)	175 (4)

Symmetry codes: (i) 
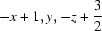
; (ii) 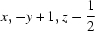.

**Fig. 1.**
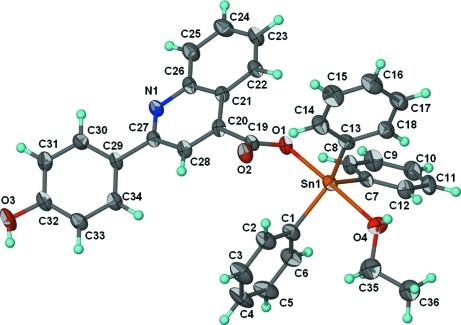
70% Probability thermal ellipsoid plot (Barbour, 2001) of Sn(C6H5)3(C2H6O)(C15H10NO). Hydrogen atoms are drawn as spheres of arbitrary radii. The disorder in the ethanol molecule is not shown.

## supplementary crystallographic information

### Experimental

Re-distilled benzaldehyde (12 ml) and pyruvic acid (11 g) were boiled in ethanol (100 ml) and to the solution was added 4-hydroxyaniline (11.5 ml). The mixture was heated for another 3 h. The solution was cooled and the solid product recrystallized from ethanol to give yellow 2-(4-hydroxyphenyl)quinoline-4-carboxylic acid.

Triphenyltin hydroxide (0.37 g, 1 mmol) and 2-(4-hydroxyphenyl)quinoline-4-carboxylic acid (0.27 g, 1 mol) were heated in ethanol (25 mol) until the reactants dissolved completely. The solution was filtered and the solvent allowed to evaporate slowly. Crystals were deposited after several days.

### Refinement

Carbon-bound H-atoms were placed in calculated positions (C—H 0.95–0.99 Å) and were included in the refinement in the riding model approximation, with *U*(H) set to 1.2–1.5*U*_eq_(C). The hydroxy H-atoms were located in a difference Fourier map, and were refined with a distance restraint of O–H 0.84±0.01 Å.

The coordinated ethanol molecule is disordered over two positions in the carbon atoms; the occupancies could not be refined, and were arbitrarily fixed as 50:50. The O–C distances were restrained to 1.45±0.01 Å and the C–C distances to 1.54±0.01 Å; the O···C distance was restrained to 2.45±0.01 Å. Additionally, the displacement parameters of the C35 atom were restrained to those of the C36 atom (and those of the C35' atom to those of the C36' atom). Restraining the temperature factors of the C35 and C35' pair of atoms to be equal led to larger peaks/deeper holes. The anisotropic displacement factors of the disordered atoms were restrained to be nearly isotropic.

The final difference Fourier map had a large peak in the vicinity of the Sn1 atom and a deep hole in the vicinity of the disordered atoms.

### Crystal data

**Table:**

[Sn(C_6_H_5_)_3_(C_16_H_10_NO_3_)(C_2_H_6_O)]	*F*(000) = 2688
*M**_r_* = 660.31	*D*_x_ = 1.450 Mg m^−^^3^
Monoclinic, *C*2/*c*	Mo *K*α radiation, λ = 0.71073 Å
Hall symbol: -C 2yc	Cell parameters from 7502 reflections
*a* = 38.9542 (5) Å	θ = 2.3–27.9°
*b* = 9.7259 (2) Å	µ = 0.89 mm^−^^1^
*c* = 17.8594 (3) Å	*T* = 100 K
β = 116.632 (1)°	Irregular block, yellow
*V* = 6048.4 (2) Å^3^	0.30 × 0.20 × 0.10 mm
*Z* = 8	

### Data collection

**Table:**

Bruker SMART APEX diffractometer	6937 independent reflections
Radiation source: fine-focus sealed tube	5517 reflections with *I* > 2σ(*I*)
graphite	*R*_int_ = 0.038
ω scans	θ_max_ = 27.5°, θ_min_ = 1.2°
Absorption correction: multi-scan (*SADABS*; Sheldrick, 1996)	*h* = −50→50
*T*_min_ = 0.777, *T*_max_ = 0.917	*k* = −12→12
23944 measured reflections	*l* = −22→23

### Refinement

**Table:**

Refinement on *F*^2^	Primary atom site location: structure-invariant direct methods
Least-squares matrix: full	Secondary atom site location: difference Fourier map
*R*[*F*^2^ > 2σ(*F*^2^)] = 0.036	Hydrogen site location: inferred from neighbouring sites
*wR*(*F*^2^) = 0.104	H atoms treated by a mixture of independent and constrained refinement
*S* = 1.26	*w* = 1/[σ^2^(*F*_o_^2^) + (0.05*P*)^2^] where *P* = (*F*_o_^2^ + 2*F*_c_^2^)/3
6937 reflections	(Δ/σ)_max_ = 0.001
395 parameters	Δρ_max_ = 1.21 e Å^−^^3^
19 restraints	Δρ_min_ = −1.30 e Å^−^^3^

### Special details

**Table:**

Geometry. All e.s.d.'s (except the e.s.d. in the dihedral angle between two l.s. planes) are estimated using the full covariance matrix. The cell e.s.d.'s are taken into account individually in the estimation of e.s.d.'s in distances, angles and torsion angles; correlations between e.s.d.'s in cell parameters are only used when they are defined by crystal symmetry. An approximate (isotropic) treatment of cell e.s.d.'s is used for estimating e.s.d.'s involving l.s. planes.

### Fractional atomic coordinates and isotropic or equivalent isotropic displacement parameters (Å^2^)

**Table:**

	*x*	*y*	*z*	*U*_iso_*/*U*_eq_	Occ. (<1)
Sn1	0.644792 (5)	0.66381 (2)	0.651964 (13)	0.01976 (8)	
O1	0.64978 (6)	0.4794 (2)	0.72684 (13)	0.0230 (5)	
O2	0.58946 (6)	0.4207 (2)	0.64055 (13)	0.0259 (5)	
O3	0.46240 (7)	0.2226 (3)	0.92855 (17)	0.0363 (6)	
H3	0.4486 (14)	0.294 (4)	0.913 (4)	0.09 (2)*	
O4	0.63966 (7)	0.8677 (2)	0.57574 (15)	0.0275 (5)	
H4	0.6351 (11)	0.861 (4)	0.5250 (10)	0.040 (12)*	
N1	0.61942 (7)	0.1541 (3)	0.90533 (16)	0.0210 (5)	
C1	0.59940 (9)	0.7431 (3)	0.6739 (2)	0.0257 (7)	
C2	0.56082 (9)	0.7165 (4)	0.6215 (2)	0.0314 (8)	
H2	0.5539	0.6600	0.5735	0.038*	
C3	0.53252 (10)	0.7721 (5)	0.6392 (3)	0.0429 (10)	
H3A	0.5062	0.7548	0.6028	0.051*	
C4	0.54256 (12)	0.8532 (4)	0.7101 (3)	0.0466 (11)	
H4A	0.5231	0.8899	0.7224	0.056*	
C5	0.58029 (11)	0.8807 (4)	0.7621 (2)	0.0414 (9)	
H5	0.5869	0.9370	0.8101	0.050*	
C6	0.60898 (10)	0.8257 (4)	0.7446 (2)	0.0324 (8)	
H6	0.6352	0.8445	0.7809	0.039*	
C7	0.70093 (8)	0.7263 (3)	0.74125 (19)	0.0210 (6)	
C8	0.71841 (9)	0.6630 (3)	0.8197 (2)	0.0254 (7)	
H8	0.7049	0.5944	0.8337	0.030*	
C9	0.75550 (10)	0.6996 (4)	0.8775 (2)	0.0329 (8)	
H9	0.7670	0.6560	0.9308	0.040*	
C10	0.77560 (9)	0.7981 (4)	0.8582 (2)	0.0321 (8)	
H10	0.8009	0.8222	0.8977	0.038*	
C11	0.75878 (10)	0.8613 (4)	0.7815 (2)	0.0310 (8)	
H11	0.7726	0.9292	0.7679	0.037*	
C12	0.72155 (9)	0.8269 (3)	0.7230 (2)	0.0275 (7)	
H12	0.7102	0.8724	0.6704	0.033*	
C13	0.64468 (9)	0.5595 (3)	0.54688 (19)	0.0218 (6)	
C14	0.61920 (9)	0.4569 (4)	0.5032 (2)	0.0289 (7)	
H14	0.5990	0.4338	0.5170	0.035*	
C15	0.62271 (11)	0.3867 (4)	0.4391 (2)	0.0372 (8)	
H15	0.6048	0.3167	0.4090	0.045*	
C16	0.65208 (10)	0.4186 (4)	0.4192 (2)	0.0360 (8)	
H16	0.6547	0.3696	0.3761	0.043*	
C17	0.67774 (10)	0.5222 (4)	0.4621 (2)	0.0308 (8)	
H17	0.6978	0.5452	0.4480	0.037*	
C18	0.67411 (9)	0.5921 (3)	0.5256 (2)	0.0253 (7)	
H18	0.6918	0.6630	0.5550	0.030*	
C19	0.61924 (8)	0.4091 (3)	0.70774 (19)	0.0207 (6)	
C20	0.62011 (8)	0.3120 (3)	0.77441 (19)	0.0208 (6)	
C21	0.65106 (8)	0.2215 (3)	0.81930 (19)	0.0201 (6)	
C22	0.68306 (9)	0.2038 (3)	0.8023 (2)	0.0245 (7)	
H22	0.6847	0.2558	0.7589	0.029*	
C23	0.71155 (9)	0.1129 (4)	0.8476 (2)	0.0297 (7)	
H23	0.7328	0.1021	0.8355	0.036*	
C24	0.70965 (10)	0.0351 (4)	0.9119 (2)	0.0329 (8)	
H24	0.7296	−0.0282	0.9429	0.039*	
C25	0.67905 (9)	0.0498 (4)	0.9305 (2)	0.0300 (7)	
H25	0.6781	−0.0028	0.9743	0.036*	
C26	0.64924 (8)	0.1431 (3)	0.8845 (2)	0.0222 (7)	
C27	0.59012 (8)	0.2362 (3)	0.85988 (19)	0.0203 (6)	
C28	0.58973 (8)	0.3161 (3)	0.79347 (19)	0.0218 (6)	
H28	0.5683	0.3731	0.7618	0.026*	
C29	0.55701 (8)	0.2372 (3)	0.87918 (19)	0.0206 (6)	
C30	0.54731 (9)	0.1182 (3)	0.9091 (2)	0.0242 (7)	
H30	0.5627	0.0382	0.9189	0.029*	
C31	0.51552 (9)	0.1148 (4)	0.9249 (2)	0.0273 (7)	
H31	0.5091	0.0327	0.9445	0.033*	
C32	0.49321 (9)	0.2316 (4)	0.9118 (2)	0.0262 (7)	
C33	0.50289 (9)	0.3521 (3)	0.8844 (2)	0.0254 (7)	
H33	0.4881	0.4329	0.8772	0.030*	
C34	0.53440 (8)	0.3541 (3)	0.8673 (2)	0.0236 (7)	
H34	0.5406	0.4363	0.8472	0.028*	
C35	0.6218 (2)	0.9877 (7)	0.5981 (5)	0.0383 (12)	0.50
H35A	0.6321	0.9950	0.6597	0.046*	0.50
H35B	0.5936	0.9756	0.5736	0.046*	0.50
C36	0.6315 (2)	1.1177 (6)	0.5627 (5)	0.0383 (12)	0.50
H36A	0.6593	1.1214	0.5809	0.057*	0.50
H36B	0.6236	1.1992	0.5832	0.057*	0.50
H36C	0.6180	1.1155	0.5014	0.057*	0.50
C35'	0.6461 (3)	1.0012 (8)	0.6165 (8)	0.084 (3)	0.50
H35C	0.6616	1.0586	0.5973	0.101*	0.50
H35D	0.6610	0.9883	0.6778	0.101*	0.50
C36'	0.6073 (3)	1.0809 (9)	0.5981 (8)	0.084 (3)	0.50
H36D	0.5961	1.1184	0.5412	0.126*	0.50
H36E	0.6128	1.1561	0.6384	0.126*	0.50
H36F	0.5890	1.0170	0.6034	0.126*	0.50

### Atomic displacement parameters (Å^2^)

**Table:**

	*U*^11^	*U*^22^	*U*^33^	*U*^12^	*U*^13^	*U*^23^
Sn1	0.01769 (11)	0.02596 (12)	0.01585 (12)	0.00055 (8)	0.00772 (9)	−0.00158 (9)
O1	0.0202 (10)	0.0300 (12)	0.0210 (11)	−0.0031 (9)	0.0112 (9)	0.0010 (9)
O2	0.0216 (11)	0.0377 (13)	0.0186 (12)	−0.0011 (10)	0.0090 (9)	0.0034 (10)
O3	0.0231 (12)	0.0563 (17)	0.0374 (15)	0.0086 (12)	0.0206 (11)	0.0141 (13)
O4	0.0355 (13)	0.0270 (12)	0.0168 (12)	0.0026 (10)	0.0088 (10)	−0.0001 (10)
N1	0.0198 (12)	0.0272 (14)	0.0177 (13)	0.0009 (10)	0.0098 (11)	0.0007 (11)
C1	0.0254 (16)	0.0329 (18)	0.0207 (17)	0.0053 (13)	0.0120 (13)	0.0011 (14)
C2	0.0283 (17)	0.0400 (19)	0.0254 (19)	0.0053 (15)	0.0116 (15)	−0.0015 (16)
C3	0.0260 (18)	0.063 (3)	0.039 (2)	0.0108 (18)	0.0136 (17)	0.002 (2)
C4	0.040 (2)	0.064 (3)	0.046 (3)	0.0192 (19)	0.029 (2)	0.001 (2)
C5	0.047 (2)	0.049 (2)	0.032 (2)	0.0128 (19)	0.0218 (18)	−0.0069 (18)
C6	0.0304 (17)	0.040 (2)	0.0262 (19)	0.0037 (15)	0.0122 (15)	−0.0052 (16)
C7	0.0182 (14)	0.0276 (16)	0.0181 (16)	−0.0013 (12)	0.0090 (12)	−0.0032 (13)
C8	0.0236 (15)	0.0324 (17)	0.0217 (17)	−0.0029 (13)	0.0115 (13)	0.0029 (14)
C9	0.0264 (17)	0.044 (2)	0.0235 (19)	−0.0006 (15)	0.0063 (14)	0.0029 (16)
C10	0.0207 (15)	0.0377 (19)	0.035 (2)	−0.0045 (14)	0.0094 (15)	−0.0077 (16)
C11	0.0264 (17)	0.0342 (19)	0.037 (2)	−0.0086 (14)	0.0182 (16)	−0.0053 (16)
C12	0.0286 (16)	0.0325 (18)	0.0212 (17)	−0.0011 (14)	0.0110 (14)	0.0020 (14)
C13	0.0220 (14)	0.0271 (16)	0.0169 (15)	0.0054 (12)	0.0092 (12)	0.0024 (13)
C14	0.0293 (17)	0.0385 (19)	0.0226 (18)	−0.0019 (14)	0.0151 (14)	−0.0039 (15)
C15	0.038 (2)	0.047 (2)	0.027 (2)	−0.0056 (17)	0.0145 (16)	−0.0123 (17)
C16	0.042 (2)	0.049 (2)	0.0227 (19)	0.0059 (17)	0.0189 (16)	−0.0024 (17)
C17	0.0330 (17)	0.040 (2)	0.0254 (18)	0.0068 (15)	0.0185 (15)	0.0036 (15)
C18	0.0246 (15)	0.0306 (18)	0.0215 (17)	0.0019 (13)	0.0109 (13)	0.0026 (14)
C19	0.0200 (14)	0.0265 (16)	0.0186 (16)	0.0007 (12)	0.0115 (13)	−0.0006 (13)
C20	0.0187 (14)	0.0267 (16)	0.0165 (15)	−0.0056 (12)	0.0074 (12)	−0.0031 (12)
C21	0.0192 (14)	0.0249 (15)	0.0163 (15)	−0.0022 (12)	0.0082 (12)	−0.0016 (13)
C22	0.0242 (15)	0.0318 (17)	0.0212 (17)	0.0004 (13)	0.0136 (13)	−0.0016 (14)
C23	0.0247 (16)	0.0375 (18)	0.033 (2)	0.0010 (15)	0.0178 (15)	0.0002 (16)
C24	0.0271 (17)	0.040 (2)	0.032 (2)	0.0123 (15)	0.0131 (15)	0.0077 (16)
C25	0.0257 (16)	0.0387 (19)	0.0271 (18)	0.0065 (14)	0.0133 (14)	0.0069 (15)
C26	0.0168 (14)	0.0279 (17)	0.0219 (17)	−0.0029 (12)	0.0086 (12)	−0.0022 (13)
C27	0.0172 (14)	0.0249 (16)	0.0187 (16)	−0.0022 (12)	0.0080 (12)	−0.0009 (13)
C28	0.0161 (13)	0.0300 (17)	0.0172 (15)	−0.0005 (12)	0.0054 (12)	0.0015 (13)
C29	0.0161 (13)	0.0304 (17)	0.0168 (15)	−0.0012 (12)	0.0085 (12)	0.0009 (13)
C30	0.0233 (15)	0.0269 (16)	0.0244 (17)	0.0030 (13)	0.0125 (14)	0.0022 (14)
C31	0.0236 (16)	0.0345 (17)	0.0248 (18)	−0.0011 (14)	0.0118 (14)	0.0056 (15)
C32	0.0194 (15)	0.0396 (19)	0.0207 (17)	0.0014 (14)	0.0100 (13)	0.0022 (15)
C33	0.0192 (14)	0.0347 (18)	0.0215 (17)	0.0061 (13)	0.0085 (13)	0.0035 (14)
C34	0.0201 (14)	0.0311 (17)	0.0189 (16)	0.0018 (13)	0.0081 (13)	0.0045 (13)
C35	0.035 (3)	0.040 (3)	0.036 (3)	−0.003 (2)	0.013 (2)	−0.002 (2)
C36	0.035 (3)	0.040 (3)	0.036 (3)	−0.003 (2)	0.013 (2)	−0.002 (2)
C35'	0.065 (5)	0.032 (3)	0.109 (6)	0.013 (3)	−0.001 (4)	−0.012 (4)
C36'	0.065 (5)	0.032 (3)	0.109 (6)	0.013 (3)	−0.001 (4)	−0.012 (4)

### Geometric parameters (Å, °)

**Table:**

Sn1—C1	2.120 (3)	C16—H16	0.9500
Sn1—C13	2.131 (3)	C17—C18	1.382 (4)
Sn1—C7	2.138 (3)	C17—H17	0.9500
Sn1—O1	2.193 (2)	C18—H18	0.9500
Sn1—O4	2.363 (2)	C19—C20	1.509 (4)
O1—C19	1.279 (3)	C20—C28	1.371 (4)
O2—C19	1.245 (4)	C20—C21	1.416 (4)
O3—C32	1.363 (4)	C21—C26	1.419 (4)
O3—H3	0.846 (10)	C21—C22	1.420 (4)
O4—C35'	1.454 (7)	C22—C23	1.365 (5)
O4—C35	1.502 (6)	C22—H22	0.9500
O4—H4	0.844 (10)	C23—C24	1.405 (5)
N1—C27	1.330 (4)	C23—H23	0.9500
N1—C26	1.374 (4)	C24—C25	1.380 (4)
C1—C2	1.393 (5)	C24—H24	0.9500
C1—C6	1.399 (5)	C25—C26	1.411 (4)
C2—C3	1.386 (5)	C25—H25	0.9500
C2—H2	0.9500	C27—C28	1.412 (4)
C3—C4	1.391 (6)	C27—C29	1.477 (4)
C3—H3A	0.9500	C28—H28	0.9500
C4—C5	1.368 (6)	C29—C34	1.395 (4)
C4—H4A	0.9500	C29—C30	1.396 (4)
C5—C6	1.396 (5)	C30—C31	1.387 (4)
C5—H5	0.9500	C30—H30	0.9500
C6—H6	0.9500	C31—C32	1.385 (5)
C7—C12	1.393 (4)	C31—H31	0.9500
C7—C8	1.396 (4)	C32—C33	1.386 (5)
C8—C9	1.394 (5)	C33—C34	1.391 (4)
C8—H8	0.9500	C33—H33	0.9500
C9—C10	1.376 (5)	C34—H34	0.9500
C9—H9	0.9500	C35—C36	1.537 (7)
C10—C11	1.371 (5)	C35—H35A	0.9900
C10—H10	0.9500	C35—H35B	0.9900
C11—C12	1.396 (5)	C36—H36A	0.9800
C11—H11	0.9500	C36—H36B	0.9800
C12—H12	0.9500	C36—H36C	0.9800
C13—C14	1.377 (5)	C35'—C36'	1.595 (13)
C13—C18	1.397 (4)	C35'—H35C	0.9900
C14—C15	1.391 (5)	C35'—H35D	0.9900
C14—H14	0.9500	C36'—H36D	0.9800
C15—C16	1.377 (5)	C36'—H36E	0.9800
C15—H15	0.9500	C36'—H36F	0.9800
C16—C17	1.384 (5)		
			
C1—Sn1—C13	131.51 (12)	C17—C18—C13	120.7 (3)
C1—Sn1—C7	114.71 (12)	C17—C18—H18	119.7
C13—Sn1—C7	112.95 (11)	C13—C18—H18	119.7
C1—Sn1—O1	92.20 (10)	O2—C19—O1	124.2 (3)
C13—Sn1—O1	96.45 (10)	O2—C19—C20	119.9 (3)
C7—Sn1—O1	89.79 (10)	O1—C19—C20	115.8 (3)
C1—Sn1—O4	86.49 (11)	C28—C20—C21	119.2 (3)
C13—Sn1—O4	85.76 (10)	C28—C20—C19	117.5 (3)
C7—Sn1—O4	89.14 (10)	C21—C20—C19	123.3 (3)
O1—Sn1—O4	177.78 (8)	C20—C21—C26	117.1 (3)
C19—O1—Sn1	117.18 (19)	C20—C21—C22	124.1 (3)
C32—O3—H3	111 (4)	C26—C21—C22	118.8 (3)
C35'—O4—Sn1	120.6 (6)	C23—C22—C21	120.7 (3)
C35—O4—Sn1	115.3 (3)	C23—C22—H22	119.7
C35'—O4—H4	121 (3)	C21—C22—H22	119.7
C35—O4—H4	116 (3)	C22—C23—C24	120.4 (3)
Sn1—O4—H4	119 (3)	C22—C23—H23	119.8
C27—N1—C26	118.7 (3)	C24—C23—H23	119.8
C2—C1—C6	118.9 (3)	C25—C24—C23	120.6 (3)
C2—C1—Sn1	123.1 (2)	C25—C24—H24	119.7
C6—C1—Sn1	117.9 (2)	C23—C24—H24	119.7
C3—C2—C1	120.2 (3)	C24—C25—C26	120.0 (3)
C3—C2—H2	119.9	C24—C25—H25	120.0
C1—C2—H2	119.9	C26—C25—H25	120.0
C2—C3—C4	120.1 (4)	N1—C26—C25	117.8 (3)
C2—C3—H3A	120.0	N1—C26—C21	122.7 (3)
C4—C3—H3A	120.0	C25—C26—C21	119.5 (3)
C5—C4—C3	120.5 (3)	N1—C27—C28	121.7 (3)
C5—C4—H4A	119.7	N1—C27—C29	117.5 (3)
C3—C4—H4A	119.7	C28—C27—C29	120.7 (3)
C4—C5—C6	119.8 (4)	C20—C28—C27	120.5 (3)
C4—C5—H5	120.1	C20—C28—H28	119.8
C6—C5—H5	120.1	C27—C28—H28	119.8
C5—C6—C1	120.4 (3)	C34—C29—C30	118.2 (3)
C5—C6—H6	119.8	C34—C29—C27	121.9 (3)
C1—C6—H6	119.8	C30—C29—C27	119.9 (3)
C12—C7—C8	118.1 (3)	C31—C30—C29	121.1 (3)
C12—C7—Sn1	122.0 (2)	C31—C30—H30	119.4
C8—C7—Sn1	119.9 (2)	C29—C30—H30	119.4
C9—C8—C7	120.6 (3)	C32—C31—C30	119.7 (3)
C9—C8—H8	119.7	C32—C31—H31	120.1
C7—C8—H8	119.7	C30—C31—H31	120.1
C10—C9—C8	120.6 (3)	O3—C32—C31	117.3 (3)
C10—C9—H9	119.7	O3—C32—C33	122.5 (3)
C8—C9—H9	119.7	C31—C32—C33	120.2 (3)
C11—C10—C9	119.5 (3)	C32—C33—C34	119.8 (3)
C11—C10—H10	120.3	C32—C33—H33	120.1
C9—C10—H10	120.3	C34—C33—H33	120.1
C10—C11—C12	120.7 (3)	C33—C34—C29	120.9 (3)
C10—C11—H11	119.6	C33—C34—H34	119.5
C12—C11—H11	119.6	C29—C34—H34	119.5
C7—C12—C11	120.5 (3)	O4—C35—C36	107.4 (5)
C7—C12—H12	119.7	O4—C35—H35A	110.2
C11—C12—H12	119.7	C36—C35—H35A	110.2
C14—C13—C18	118.8 (3)	O4—C35—H35B	110.2
C14—C13—Sn1	124.1 (2)	C36—C35—H35B	110.2
C18—C13—Sn1	117.0 (2)	H35A—C35—H35B	108.5
C13—C14—C15	120.7 (3)	O4—C35'—C36'	113.3 (8)
C13—C14—H14	119.7	O4—C35'—H35C	108.9
C15—C14—H14	119.7	C36'—C35'—H35C	108.9
C16—C15—C14	120.1 (3)	O4—C35'—H35D	108.9
C16—C15—H15	119.9	C36'—C35'—H35D	108.9
C14—C15—H15	119.9	H35C—C35'—H35D	107.7
C15—C16—C17	119.9 (3)	C35'—C36'—H36D	109.5
C15—C16—H16	120.1	C35'—C36'—H36E	109.5
C17—C16—H16	120.1	H36D—C36'—H36E	109.5
C18—C17—C16	119.9 (3)	C35'—C36'—H36F	109.5
C18—C17—H17	120.0	H36D—C36'—H36F	109.5
C16—C17—H17	120.0	H36E—C36'—H36F	109.5
			
C1—Sn1—O1—C19	53.6 (2)	C14—C15—C16—C17	−1.1 (6)
C13—Sn1—O1—C19	−78.6 (2)	C15—C16—C17—C18	0.9 (5)
C7—Sn1—O1—C19	168.4 (2)	C16—C17—C18—C13	−0.2 (5)
C1—Sn1—O4—C35'	61.0 (6)	C14—C13—C18—C17	−0.3 (5)
C13—Sn1—O4—C35'	−166.9 (6)	Sn1—C13—C18—C17	175.4 (2)
C7—Sn1—O4—C35'	−53.8 (6)	Sn1—O1—C19—O2	15.7 (4)
C1—Sn1—O4—C35	23.0 (4)	Sn1—O1—C19—C20	−161.42 (19)
C13—Sn1—O4—C35	155.1 (4)	O2—C19—C20—C28	−46.6 (4)
C7—Sn1—O4—C35	−91.8 (4)	O1—C19—C20—C28	130.7 (3)
C13—Sn1—C1—C2	9.9 (4)	O2—C19—C20—C21	135.5 (3)
C7—Sn1—C1—C2	178.5 (3)	O1—C19—C20—C21	−47.2 (4)
O1—Sn1—C1—C2	−90.7 (3)	C28—C20—C21—C26	−2.3 (4)
O4—Sn1—C1—C2	91.1 (3)	C19—C20—C21—C26	175.5 (3)
C13—Sn1—C1—C6	−170.3 (2)	C28—C20—C21—C22	176.8 (3)
C7—Sn1—C1—C6	−1.7 (3)	C19—C20—C21—C22	−5.3 (5)
O1—Sn1—C1—C6	89.1 (3)	C20—C21—C22—C23	−179.0 (3)
O4—Sn1—C1—C6	−89.1 (3)	C26—C21—C22—C23	0.1 (5)
C6—C1—C2—C3	0.5 (5)	C21—C22—C23—C24	0.0 (5)
Sn1—C1—C2—C3	−179.7 (3)	C22—C23—C24—C25	−0.3 (6)
C1—C2—C3—C4	−0.9 (6)	C23—C24—C25—C26	0.3 (5)
C2—C3—C4—C5	1.0 (7)	C27—N1—C26—C25	−176.6 (3)
C3—C4—C5—C6	−0.7 (7)	C27—N1—C26—C21	3.5 (4)
C4—C5—C6—C1	0.2 (6)	C24—C25—C26—N1	180.0 (3)
C2—C1—C6—C5	−0.1 (5)	C24—C25—C26—C21	−0.2 (5)
Sn1—C1—C6—C5	−180.0 (3)	C20—C21—C26—N1	−1.0 (4)
C1—Sn1—C7—C12	−103.7 (3)	C22—C21—C26—N1	179.8 (3)
C13—Sn1—C7—C12	67.1 (3)	C20—C21—C26—C25	179.1 (3)
O1—Sn1—C7—C12	164.0 (3)	C22—C21—C26—C25	−0.1 (5)
O4—Sn1—C7—C12	−17.9 (3)	C26—N1—C27—C28	−2.7 (4)
C1—Sn1—C7—C8	78.2 (3)	C26—N1—C27—C29	175.0 (3)
C13—Sn1—C7—C8	−111.0 (3)	C21—C20—C28—C27	3.2 (5)
O1—Sn1—C7—C8	−14.1 (2)	C19—C20—C28—C27	−174.8 (3)
O4—Sn1—C7—C8	163.9 (3)	N1—C27—C28—C20	−0.7 (5)
C12—C7—C8—C9	−0.3 (5)	C29—C27—C28—C20	−178.3 (3)
Sn1—C7—C8—C9	177.9 (3)	N1—C27—C29—C34	149.5 (3)
C7—C8—C9—C10	−0.3 (5)	C28—C27—C29—C34	−32.8 (4)
C8—C9—C10—C11	0.4 (5)	N1—C27—C29—C30	−31.4 (4)
C9—C10—C11—C12	0.2 (5)	C28—C27—C29—C30	146.3 (3)
C8—C7—C12—C11	0.9 (5)	C34—C29—C30—C31	1.5 (5)
Sn1—C7—C12—C11	−177.3 (2)	C27—C29—C30—C31	−177.6 (3)
C10—C11—C12—C7	−0.8 (5)	C29—C30—C31—C32	−0.9 (5)
C1—Sn1—C13—C14	−43.2 (3)	C30—C31—C32—O3	−179.8 (3)
C7—Sn1—C13—C14	148.0 (3)	C30—C31—C32—C33	−0.9 (5)
O1—Sn1—C13—C14	55.5 (3)	O3—C32—C33—C34	−179.1 (3)
O4—Sn1—C13—C14	−124.7 (3)	C31—C32—C33—C34	2.1 (5)
C1—Sn1—C13—C18	141.4 (2)	C32—C33—C34—C29	−1.4 (5)
C7—Sn1—C13—C18	−27.4 (3)	C30—C29—C34—C33	−0.3 (5)
O1—Sn1—C13—C18	−119.9 (2)	C27—C29—C34—C33	178.8 (3)
O4—Sn1—C13—C18	59.9 (2)	C35'—O4—C35—C36	55.8 (11)
C18—C13—C14—C15	0.1 (5)	Sn1—O4—C35—C36	163.7 (4)
Sn1—C13—C14—C15	−175.3 (3)	C35—O4—C35'—C36'	−10.5 (8)
C13—C14—C15—C16	0.6 (6)	Sn1—O4—C35'—C36'	−101.1 (9)

### Hydrogen-bond geometry (Å, °)

**Table:**

*D*—H···*A*	*D*—H	H···*A*	*D*···*A*	*D*—H···*A*
O3—H3···O2^i^	0.85 (1)	1.83 (2)	2.661 (3)	166 (6)
O4—H4···N1^ii^	0.84 (1)	1.95 (1)	2.789 (4)	175 (4)

Symmetry codes: (i) −*x*+1, *y*, −*z*+3/2; (ii) *x*, −*y*+1, *z*−1/2.
